# Supplementary material for: Quatsomes Loaded with Squaraine Dye as an Effective Photosensitizer for Photodynamic Therapy
Source: Pharmaceutics. 2023 Mar 10;15(3):902. doi: 10.3390/pharmaceutics15030902 (PMC10057727; doi:10.3390/pharmaceutics15030902)
Supplement: Supplementary file 1 [file pharmaceutics-15-00902-s001.zip › pharmaceutics-2232887-supplementary.pdf]

SUPPORTING INFORMATION

Article

Quatsomes loaded with squaraine dye as an effective photosensitizer for Photodynamic Therapy

Nicolò Bordignon, [1,2] **Mariana Köber,**[3,1] **Giorgia Chinigò,**[2] **Carlotta Pontremoli,**[4] **Ettore Sansone,**[2] **Guillem Vargas-Nadal,**[1,3] **Maria Jesus Moran Plata,**[4] **Alessandra Fiorio Pla,**[2] **Nadia Barbero,***[,4] **Judit Morla-Folch,***[,1] **Nora Ventosa***[,1,3]

Figure S1. Preliminary data with Br-Sq-C4 2

Figure S2. ^{1}H NMR spectrum of Br-Sq-C12 in CDCl3. 3

Figure S3. ESI-MS spectrum of Br-Sq-C12 4

Figure S4. QS stability after 18 months 5

Figure S5. Maximum absorbance peak over time of Br-Sq-C12-loaded QS 6

Figure S6. Cell viability assays with different concentrations of Br-Sq-12 7

Preparation of dye-loaded Chol/Stk QS by DELOS-susp 8

Table S1. Detailed reagents for the preparation of the QS 8

DLS systematic study

Figure S7. Absorption of Br-Sq-12 9

Figure S8. Hydrodynamic diameter and PDI variation related to dilution 10

Figure S9. Comparison at two dilutions 11

References 12

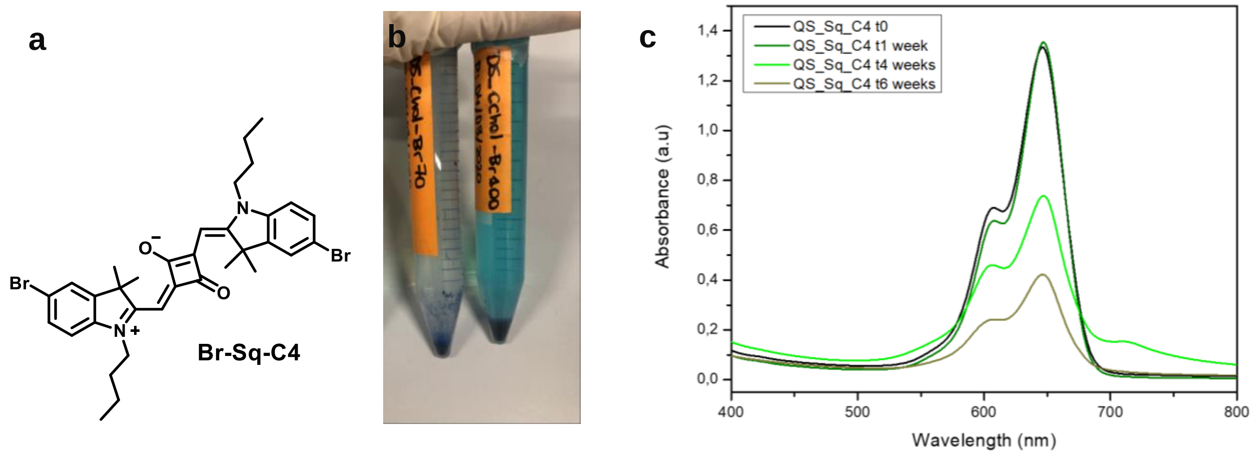

Figure S1 – Preliminary data obtained with Quatsomes (Stk/Chol) loaded with Br-Sq-C4. Representation of the chemical structure of Br-Sq-C4 (**a**). Image of two different samples of QS_Sq_C4 after 2 months from preparation (**b**). Significant Br-Sq-C4 leaking is observed from the blue precipitate. Absorption spectra over time from one sample of QS_Sq_C4 initially loaded with 100 μM. The concentration of Br-Sq-C4 at the QS after 6 weeks was less than 15 μM. (**c**)

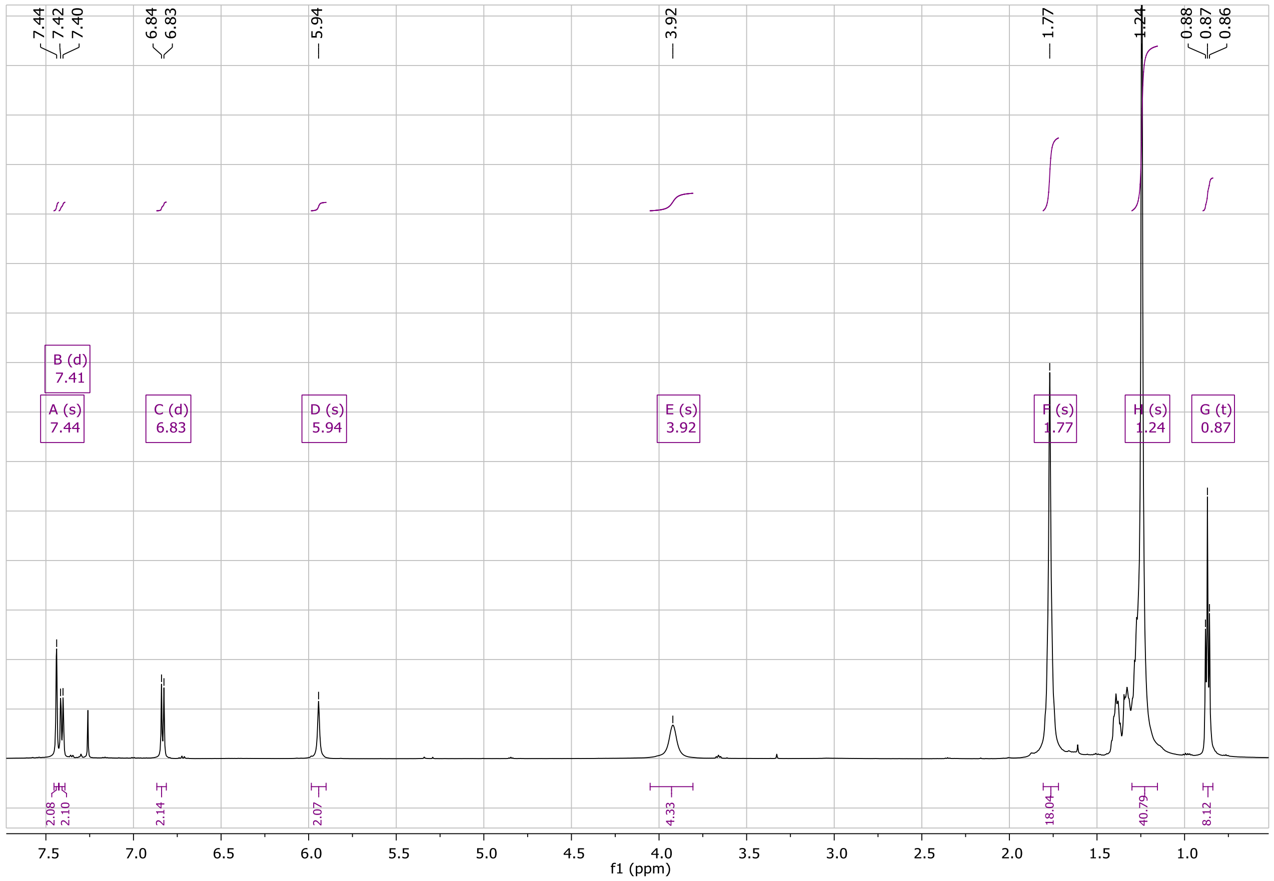

Figure S2 – ^{1}H NMR spectrum of Br-Sq-C12 in $CDCl_3$.

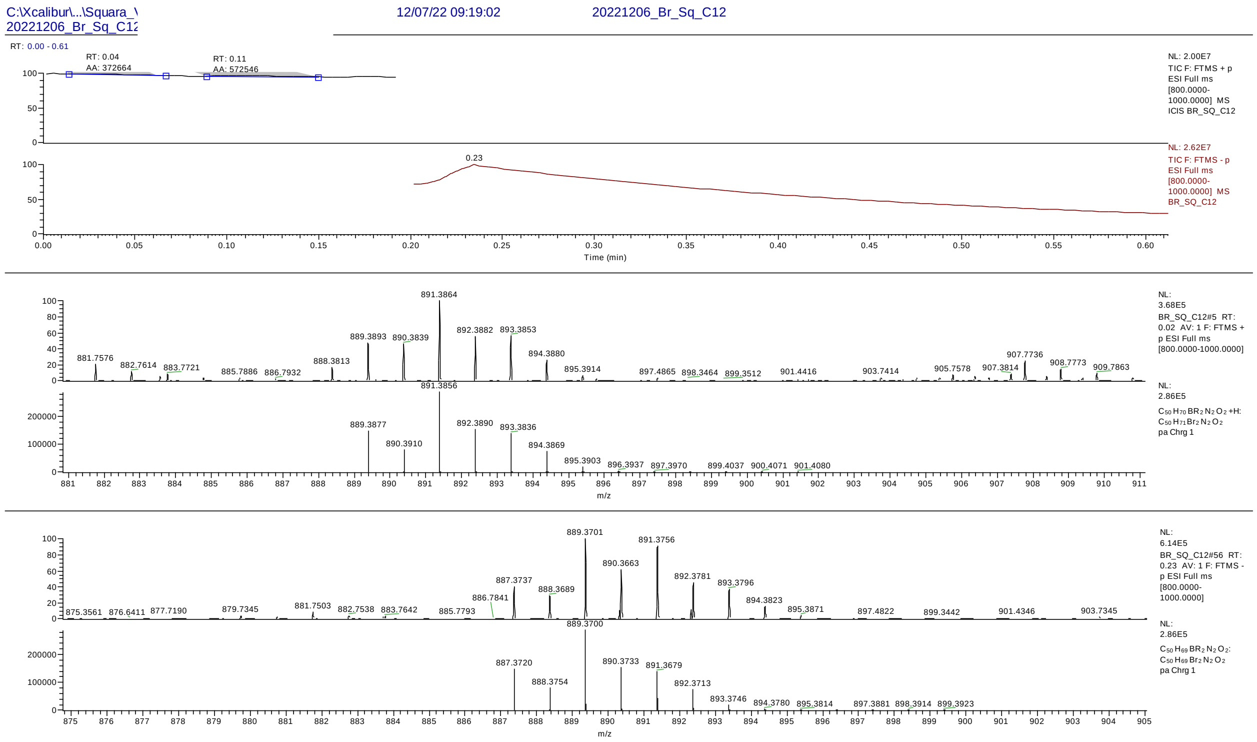

Figure S3 – ESI-MS spectrum of Br-Sq-C12.

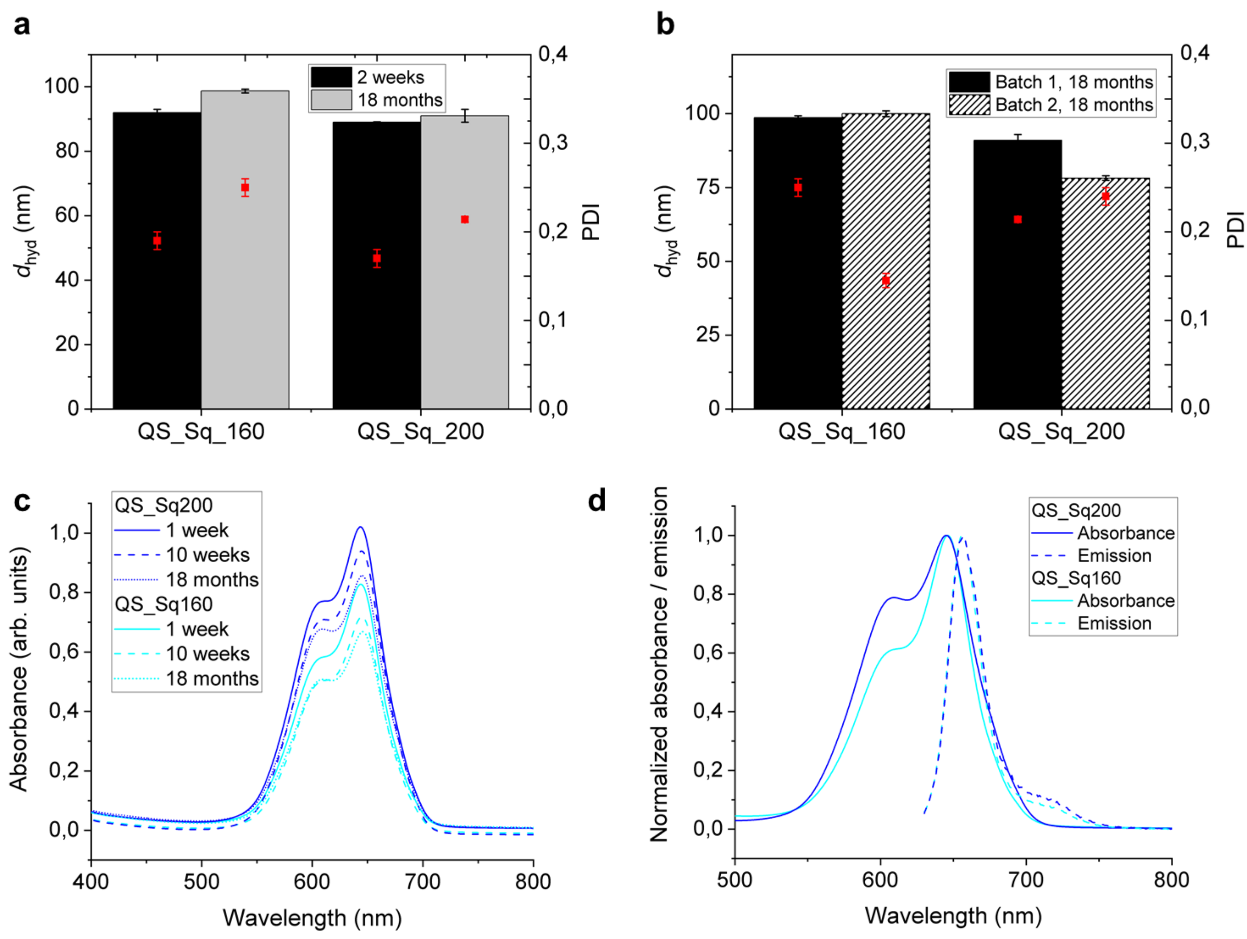

Figure S4 - QS stability after 18 months. DLS bar plot, showing hydrodynamic diameters as bars and PDI as dots, showing the very small changes in size over 18 months (a). Batch-to-batch variation in size (bars) and PDI (dots) of aged samples (18 months) (b). Absorbance spectra at 1 week, 10 weeks and 18 months (c). Normalized absorbance and emission spectra of aged samples at 18 months (d).

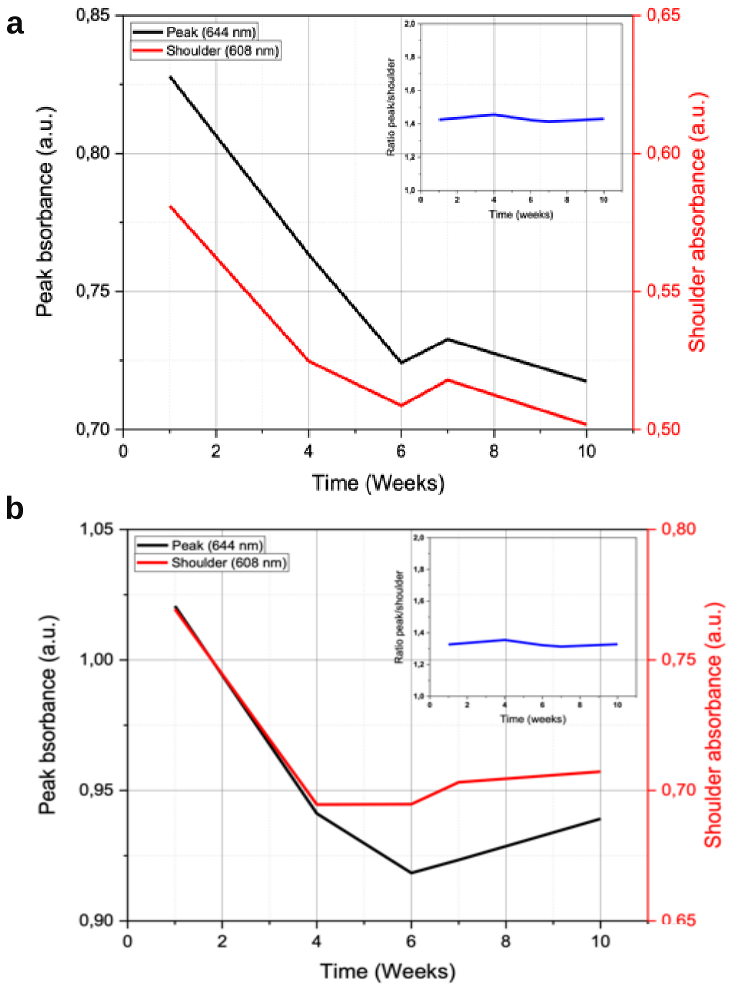

Figure S5 – Maximum absorbance peak over time of Br-Sq-C12-loaded QS. Maximum absorbance peak and shoulder trend in time and ratio peak/shoulder trend (showed on top-right in blue) of QS_Sq_160 (**a**) and QS_Sq_200 (**b**).

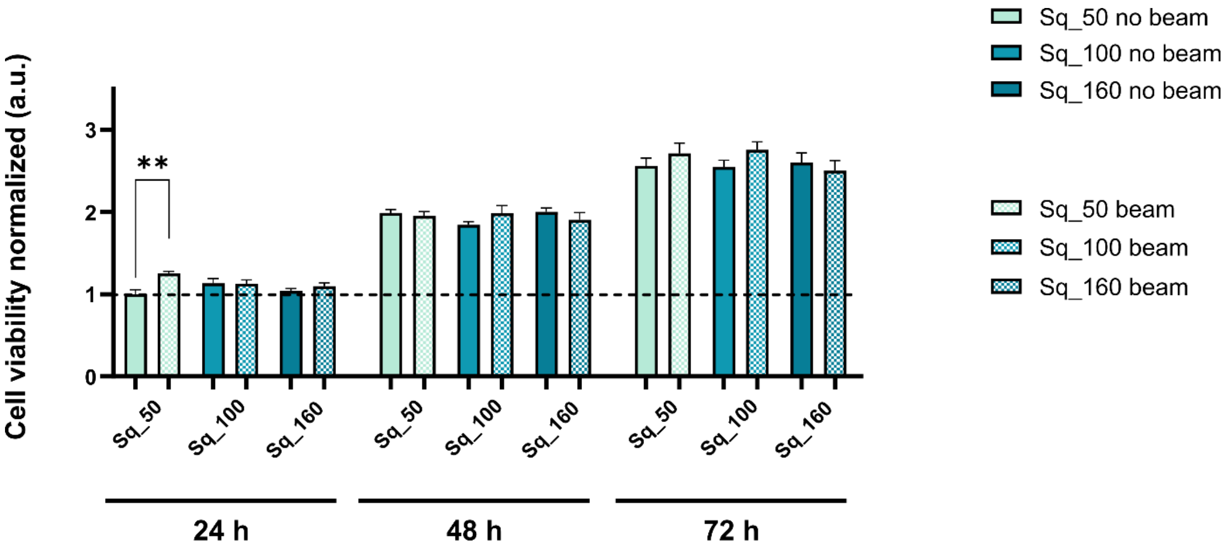

Figure S6- Cell viability assays on MCF-7 O/N treated with different concentrations of Br-Sq-12 in its free form in the dark and 24 h, 48 h, and 72 h after LED irradiation (640 nm, 7.2 J/cm^2). Data are normalized on CNTRL at 24 h (dot lines) and represented as mean ± SEM. Data refer to one representative experiment out of three. Statistical significance between irradiated and not irradiated cells (MCF-7 untreated): * P< 0.05, ** P< 0.01, *** P< 0.001, **** P< 0.0001 (t- test and Mann-Whitney test).

PREPARATION OF DYE-LOADED CHOL/STK QK BY DELOS-SUSP

The preparation of dye-loaded Quatsomes was performed using the DELOS-susp method [47,51,70]. Initially, a 6.5 mM stock solution of Br-Sq-C12 in ethanol was prepared protected from light. All the samples have the same concentration of membrane components (Cholesterol and Stk). Thus, 80.40 mg of Cholesterol (PanReac AppliChem, Castellar del Vallès, Spain) and 85.50 mg of Stearalkonium chloride (TokyoChemical Industry CO. LTD, Tokyo, Japan) (molar ratio 1:1) were dissolved in 1.8 mL ethanol (HPLC grade purity, Avantor Performance Materials Poland S.A., Silesia, Poland). Depending on the final dye concentration, 0.868 or 1.302 mL of 6.5 mM Br-Sq-C12 stock solution was subsequently added to achieve a total dye concentration of 200 and 300 μM, respectively, in the final vesicle dispersion. The final ethanol volume was adjusted to obtain 3.11 mL as the final volume. The ethanolic solution was heated to the working temperature of 38 °C and was added to the high-pressure vessel at 7.3 mL of volume. After 10 minutes of stabilization, the ethanolic solution was pressurized with CO_2 until reaching a working pressure of 11.5 MPa and obtaining a CO_2-expanded solution. After 1 h the solution was depressurized in 25.11 mL of Milli-Q water. In the depressurization step, N_2 was added to the vessel to maintain a constant pressure of 11.5 MPa inside the vessel. As shown in Table S1, the initial concentration of membrane components, meaning Stk and Chol, was in all formulations the same. After vesicle production by DELOS-susp, samples were purified by diafiltration to remove ethanol, the not-incorporated dye, and QS membrane components, using the KrosFlo® Research IIi TFF System (KR2i) (Repligen, Waltham, USA). The column in use was a 100 kDa cut-off mPES hollow fiber column (C04-E100-05-N & C02-E100-05-N, Repligen, Waltham, USA). The samples were stored at room temperature protected from light.

Table S1 – Quantities of reagents employed during the preparation of the QS samples under study.

	**Membrane components (organic phase)**				**Dispersant medium**
**Sample**	**Chol (mg)**	**Stk (mg)**	**Br-Sq-C12 from 6.5 mM stock solution (μL)**	**Ethanol (μL)**	**Water (mL)**
QS_Blank	80.4	85.5	-	3110	25.11
QS_Sq_160	80.4	85.5	868	2242	25.11
QS_Sq_200	80.4	85.5	1302	1808	25.11

SYSTEMATIC DLS STUDY

The instrument in use for DLS and ELS measurements (see materials & methods section) employs a 633 nm laser. The maximum absorbance wavelength of the dye in use is 641 nm (Figure S7).

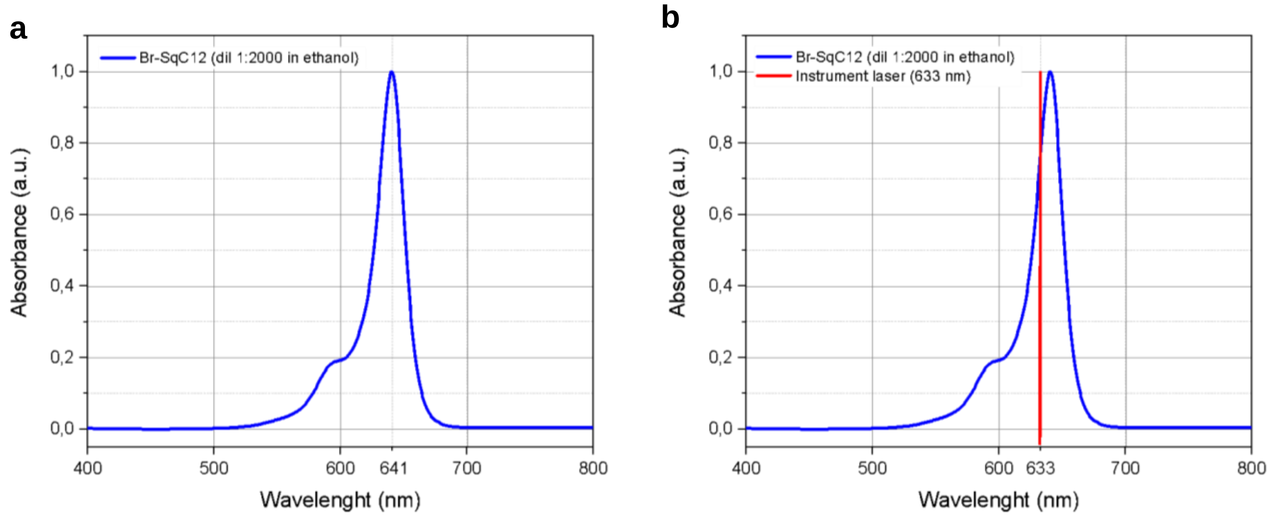

Figure S7 - Normalized Br-Sq-C12 spectrum in EtOH. Laser wavelength and maximum absorbance wavelength are reported on the x-axes. Br-Sq-C12 spectrum (a), comparison with laser wavelength (b).

Since the dye's maximum absorbance wavelength is very close to the instrument laser's wavelength, and the local concentration of dye in the vesicles in solution was considerable, the dye could absorb part of the coherent incident light and consequently emit non-coherent fluorescent light, limiting the scattering and so reducing data quality [69].

In the case of the samples reported in this work, DLS produced unreliable results compared to the ones obtained with blank vesicles presenting the same composition, measuring hydrodynamic diameter values over 100 nm.

This happened in ELS too, with z-potential values around 0 mV for both the dye-loaded samples, while the expected values were more positive, due to the presence of the positively charged Stearalkonium as a membrane component of the vesicles.

An experimental protocol was fine-tuned to identify the correct dilution to conduct DLS measurements and obtain reliable results, trying to reduce the concentration of the absorbing molecules in solution, and so enhancing the quality and reliability of the data.

Hydrodynamic diameters and PDI were systematically measured at progressive dilutions (1:2, 1:5, 1:10, 1:50, 1:100, 1:500, 1:1000, 1:2000, 1:10000) with the same instrument setup (see material & methods section) in order to identify a range in which their values were stable.

Enhancing the dilution rates, it was possible to see a decrease of the values, followed by a stabilization phase and then a progressive increase, due to the shortage of particles in solution and so the lack of light scattering (Figure S8). This phenomenon involved both hydrodynamic diameter and PDI values.

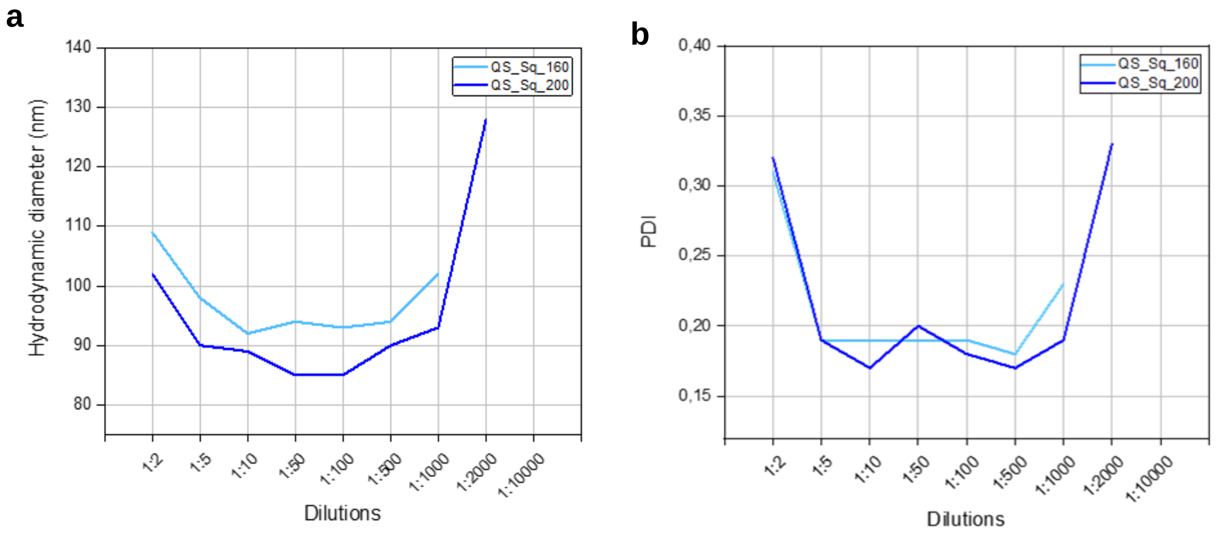

Figure S8 - Hydrodynamic diameter variation related to dilution (a). PDI variation related to dilution (b).

On the basis of the obtained results, it was considered reasonable, to obtain reliable results, to work in a range of dilutions between 1:10 and 1:100. In order to limit the experimental error due to working with very small amounts of solutions, all the DLS and ELS measurements were carried at dilution 1:10 for all the dye loaded samples.

Working at this dilution the values of hydrodynamic diameter were reliable (Figure S8a) and, moreover, they were confirmed by Cryo-TEM (as shown in the previous parts of the work); the z-potential values stabilized around the positive expected value too (Figure S8b).

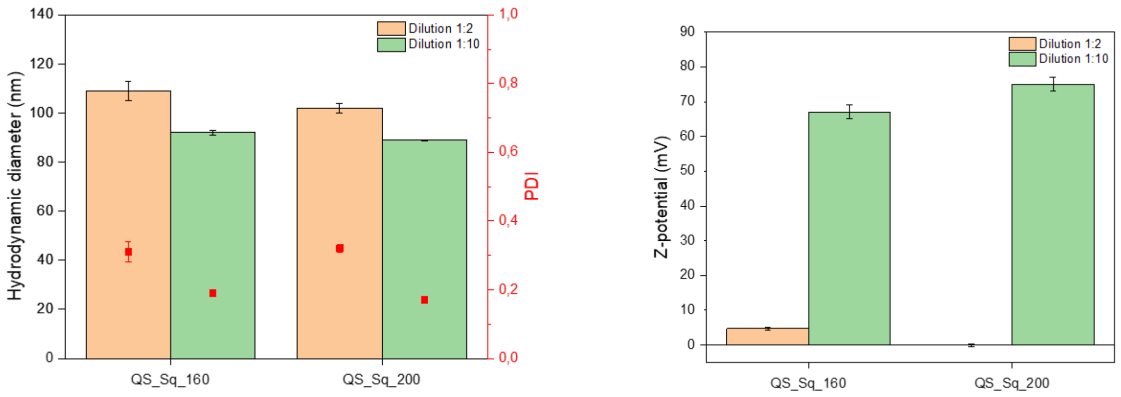

Figure S9 - Comparison of results at dilution 1:2 and 1:10 (a). Comparison of Z-potential values at dilution 1:2 and 1:10 (b).

In the bar plot is possible to observe a bigger difference between the values of hydrodynamic diameter obtained at the two different dilutions. As mentioned before, the concentration of the dye plays a crucial role in decreasing the quality of the data [69].

REFERENCES

47. Cabrera, I.; Elizondo, E.; Esteban, O.; Corchero, J.L.; Melgarejo, M.; Pulido, D.; Córdoba, A.; Moreno, E.; Unzueta, U.; Vazquez, E.; et al. Multifunctional Nanovesicle-Bioactive Conjugates Prepared by a One-Step Scalable Method Using CO2-Expanded Solvents. *Nano Lett* **2013**, *13*, 3766–3774, doi:10.1021/nl4017072
51. Ventosa, N.; Veciana, J.; Sala, S.; Cano, M. Method for Obtaining Micro- and Nano-Disperse Systems. WO 2006/079889 2005. 2006 August 3.
69. Geißler, D.; Gollwitzer, C.; Sikora, A.; Minelli, C.; Krumrey, M.; Resch-Genger, U. Effect of Fluorescent Staining on Size Measurements of Polymeric Nanoparticles Using DLS and SAXS. *Anal. Methods* **2015**, *7*, 9785–9790, doi:10.1039/C5AY02005K.
70. Elizondo, E.; Larsen, J.; Hatzakis, N.S.; Cabrera, I.; Bjørnholm, T.; Veciana, J.; Stamou, D.; Ventosa, N. Influence of the Preparation Route on the Supramolecular Organization of Lipids in a Vesicular System. *J Am Chem Soc* **2012**, *134*, 1918–1921, doi:10.1021/ja2086678.
